# Balanced neural architecture and the idling brain

**DOI:** 10.3389/fncom.2014.00056

**Published:** 2014-05-27

**Authors:** Brent Doiron, Ashok Litwin-Kumar

**Affiliations:** ^1^Department of Mathematics, University of PittsburghPittsburgh, PA, USA; ^2^Center for the Neural Basis of Cognition, University of Pittsburgh and Carnegie Mellon UniversityPittsburgh, PA, USA; ^3^Program for Neural Computation, University of Pittsburgh and Carnegie Mellon UniversityPittsburgh, PA, USA

**Keywords:** balanced cortical networks, neural variability, spontaneous cortical activity, cortical circuits, spiking models

## Abstract

A signature feature of cortical spike trains is their trial-to-trial variability. This variability is large in the spontaneous state and is reduced when cortex is driven by a stimulus or task. Models of recurrent cortical networks with unstructured, yet balanced, excitation and inhibition generate variability consistent with evoked conditions. However, these models produce spike trains which lack the long timescale fluctuations and large variability exhibited during spontaneous cortical dynamics. We propose that global network architectures which support a large number of stable states (attractor networks) allow balanced networks to capture key features of neural variability in both spontaneous and evoked conditions. We illustrate this using balanced spiking networks with clustered assembly, feedforward chain, and ring structures. By assuming that global network structure is related to stimulus preference, we show that signal correlations are related to the magnitude of correlations in the spontaneous state. Finally, we contrast the impact of stimulation on the trial-to-trial variability in attractor networks with that of strongly coupled spiking networks with chaotic firing rate instabilities, recently investigated by Ostojic (2014). We find that only attractor networks replicate an experimentally observed stimulus-induced quenching of trial-to-trial variability. In total, the comparison of the trial-variable dynamics of single neurons or neuron pairs during spontaneous and evoked activity can be a window into the global structure of balanced cortical networks.

## 1. Introduction

The rich structure of neural firing patterns provides ample challenges for the systems neuroscience community. The complexity of *in vivo* neural responses depends, in part, on detailed single neuron biophysics, synaptic dynamics, and network interactions. To make sense of such complexity, it is often necessary to treat brain activity as probabilistic, similar to how statistical physics treats large ensembles of particles. In this spirit, collecting the responses of a neuron (or a population of neurons) over many trials of an experiment permits a statistical characterization of neural activity. A widespread observation is that the trial averaged response of a neuron's spike output is stimulus or action tuned (Decharms and Zador, 2000), and this has grounded many theories of neural coding (Dayan and Abbott, 2001). However, only considering the trial averaged response of a neuron glosses over many complex response dynamics that may give further insight into neural function.

Large trial-to-trial variability of the neural response is a general characteristic of cortical dynamics (Britten et al., 1992; Shadlen and Newsome, 1994; Averbeck et al., 2006; Cohen and Kohn, 2011; Ponce-Alvarez et al., 2013). In stimulus or task evoked states, spiking responses are well modeled as an inhomogeneous Poisson process, whose firing rate dynamics can be obtained from trial averaged responses. In this case, the response variability is equal to the trial averaged evoked response, and little is learned about cortical processing by considering neural variability. Neural activity during spontaneous dynamics (dynamics in the absence of a driving stimulus or action) is more complex (Ringach, 2009). In particular, the trial-to-trial spike variability during spontaneous states is larger than that predicted by a Poisson process (Churchland et al., 2006, 2010), and exhibits fluctuations over a range of timescales (Smith and Kohn, 2008). The state of cortex during spontaneous conditions is both an influence (Arieli et al., 1996; Fukushima et al., 2012) and a reflection of stimulus evoked activity (Tsodyks et al., 1999; Luczak et al., 2009; Luczak and MacLean, 2012). Thus, understanding the mechanics behind spontaneous activity may give new insight into the structure and function of cortical circuits.

Recent theoretical studies have shown that cortical models with balanced excitation and inhibition and clustered excitatory connectivity capture the trial-to-trial variability of both spontaneous and evoked conditions (Deco and Hugues, 2012; Litwin-Kumar and Doiron, 2012). In this article, we present a unified view of spontaneous dynamics in a variety of neural architectures. The core mechanic is that networks with a large number of stable states allow spontaneous fluctuations to stochastically “sample” the various states (Goldberg et al., 2004), leading to high spiking variability in spontaneous conditions. For stimuli to quench spiking variability, such models require that the recurrent architecture that supports network metastability be coherent with feedforward afferent projections. Under this assumption, we predict that pairwise spiking correlations as measured in the spontaneous state will be related to signal correlations in evoked states. Our article extends the framework of balanced excitation and inhibition (van Vreeswijk and Sompolinsky, 1996, 1998; Renart et al., 2010) to more structured network architectures, leading to a theory of variability that is consistent with spontaneously active cortex and its relation to the evoked cortical response.

## 2. Results

### 2.1. Neural variability in balanced excitatory and inhibitory networks

**Figure 1:**
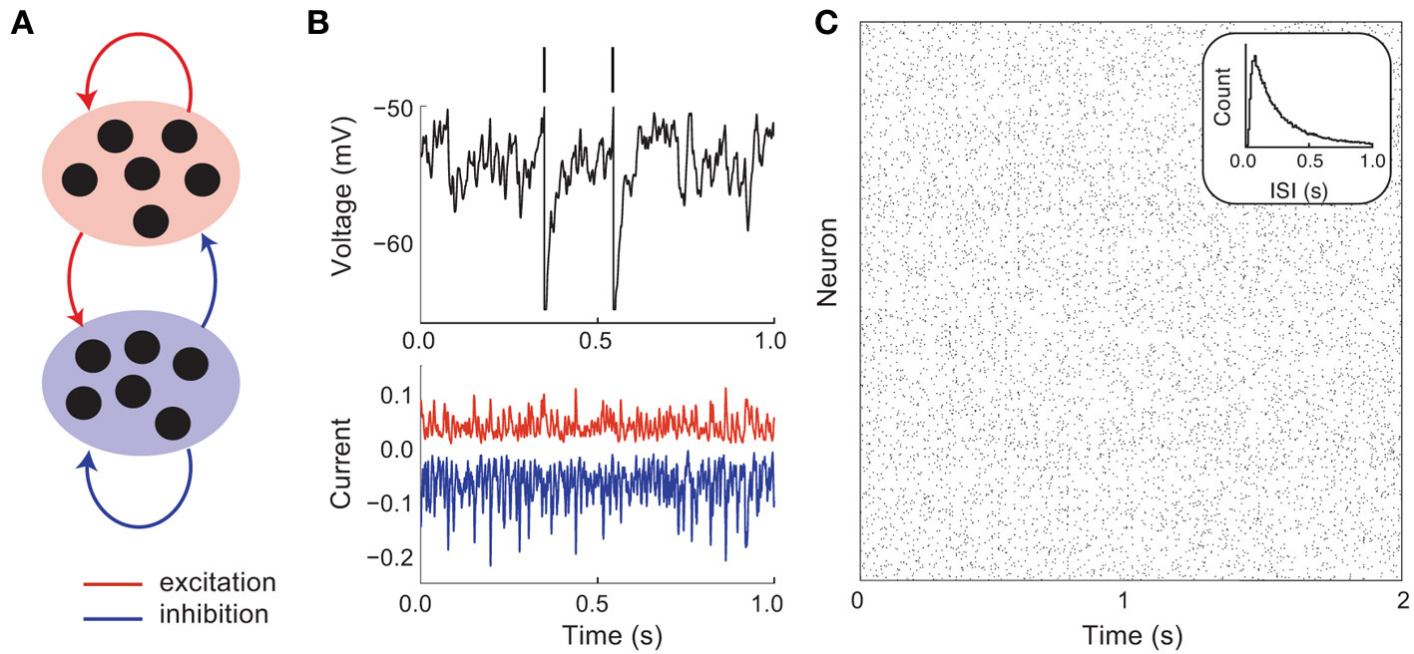
**Asynchronous and irregular dynamics in balanced excitatory and inhibitory networks. (A)** Schematic of excitatory (red) and inhibitory (blue) network. **(B)** Membrane potential dynamics (black) from a representative neuron in the network. Membrane potential threshold crossings are indicated with a vertical spike. The irregular membrane potential activity is due to large amplitude, yet opposing, excitation (red) and inhibition (blue). **(C)** Network spike time raster plot. The inter-spike interval histogram is broad with a short refractory period (see inset).

Cortical circuits have recurrent excitatory and inhibitory connections that are sparse and random (Figure 1A). As a first model, we consider no underlying global cortical architecture and rather assume wiring that obeys a pairwise independent model (meaning that a single connection between two neurons occurs with a probability that is independent of the rest of the network). Despite sparse wiring, the high number of cortical neurons ensures that a postsynaptic cell receives a large number (~10^3^) of synaptic inputs from other cortical neurons (Binzegger et al., 2004). Furthermore, the membrane potential deflection from an individual excitatory or inhibitory input can be on the order of 1 mV (Lefort et al., 2009). The convergence of a high number of large amplitude excitatory or inhibitory inputs could overwhelm a postsynaptic cell. However, in practice, the firing rates of cortical neurons are neither negligible nor driven to saturation (Hromádka et al., 2008). To explain this seeming contradiction between anatomy and physiology, Shadlen and Newsome (1994, 1998) proposed that large excitation and inhibition effectively balance one another (on average), so that the net mean input is small and output rates can be moderate (Figure 1B). This conjecture has experimental support from *in vivo* whole cell recordings from cortical neurons, in which the magnitudes of isolated excitatory and inhibitory inputs are large yet balanced when neurons are near their resting state (Haider et al., 2006).

A key feature of balanced cortical networks is that spiking activity is dominated by the *fluctuations* in synaptic input (Shadlen and Newsome, 1994). These fluctuations can be sizable, since synaptic strengths are relatively large (only a small fraction of afferent inputs are necessary to drive the voltage to its threshold value). Synaptic input fluctuations provide a mechanism for the large dynamic and trial-to-trial variability of spiking activity that is characteristic of cortex (Figure 1B, top). Using techniques from statistical physics, van Vreeswijk and Sompolinsky (1996, 1998) provided a formal link between balance in recurrent networks and spiking variability. Specifically, they considered the statistics of the synaptic input into a representative cell in the network that received on average *K* inputs from recurrent excitatory and inhibitory wiring. With minimal assumptions, the mean *M* and variance *V* of the input obey:

$$\begin{array}{l} M=J_{E}r_{E}K-J_{I}r_{I}K+I_{\text{ext}},\\ V\;={\left(J_{E}\right)}^{2}r_{E}K+{\left(J_{I}\right)}^{2}r_{I}K. \end{array}$$

Here *J*_α_ and *r*_α_ are the synaptic strength and firing rates of the α ∈ {*E*, *I*} population and *I*_ext_ is an external drive to the network. For a reasonable theory we require that *M* and *V* to be independent of system size for large *K*. For the input variance *V* to neither vanish nor explode as *K* → ∞, we must scale *J*_α_ = *j*_α_/$\sqrt{K}$ where the parameter *j*_α_ ~ *O*(1). If we let the external input scale as *I*_ext_ = *i*_ext_$\sqrt{K}$, then we have the following expression for *M*:

$$M=\sqrt{K}\underset{O\left(1/\sqrt{K}\right)}{\underset{︸}{\left(j_{E}r_{E}-j_{I}r_{I}+i_{\text{ext}}\right)}}\sim O(1).$$

For the mean input *M* to neither vanish nor explode as *K* → ∞, the interior of the bracketed term must, as a whole, scale as *O*(1/$\sqrt{K}$). This only occurs when the recurrent and external excitation are balanced by recurrent inhibition, and with *i*_ext_ >0 this can occur robustly over a wide range of network parameters.

Using this mean field approach van Vreeswijk and Sompolinsky showed that balanced networks were dynamically stable over a wide range of parameters, with asynchronous spiking dynamics (Figure 1C), and single neuron statistics consistent with Poisson-like variability (Figure 1C, inset). An important component of this variability is that it is self-generated through network interactions, making it akin to high dimensional chaos. Such chaotic network-based variability has *in vivo* experimental support (London et al., 2010), and been the focus of recent theoretical investigations (Banerjee et al., 2008; Monteforte and Wolf, 2010, 2012). Furthermore, the fact that the variability is self-generated shows that the high degree of variability of cortical dynamics may be an intrinsic property of a cortical circuit, rather than being inherited from external sources to the network.

In these analyses it is often assumed that connectivity is sparse. However, later work by Renart et al. (2010) relaxed this assumption and showed that asynchronous activity could be achieved in densely coupled balanced networks. More recent work has discussed the impact of balance in working memory (Boerlin and Denève, 2011; Lim and Goldman, 2013)^1^, and the formation of orientation selectivity in visual cortices that lack functional topography (Hansel and van Vreeswijk, 2012). In total, balanced networks have been a successful framework to probe the mechanisms through which cortical dynamics are irregular, both in time and over repeated presentations of a fixed input.

### 2.2. Spontaneous and evoked dynamics in metastable population dynamics

The spike patterns from model neurons in balanced networks capture the irregular and asynchronous spike dynamics of evoked cortical response. However, balanced networks fail to capture the dynamics of cortical networks in spontaneous conditions (i.e., when the cortex is “idling”). In both sensory and motor cortices, the variability of spiking activity during spontaneous conditions is larger than that of a homogeneous Poisson process of the same firing rate (Churchland et al., 2006, 2010). A useful simplification is to treat a spike train in spontaneous conditions as a “doubly stochastic” process (Byron et al., 2009; Churchland and Abbott, 2012; Ponce-Alvarez et al., 2013), with one process modeling fast Poisson-like spike discharge and the other process capturing a slow fluctuation in the firing rate. Balanced networks of model neurons with independent wiring do not show the slow firing rate variability, and rather only capture the fast spiking variability (Figure 1C). In this section we show how higher order architectural structure in excitatory–excitatory connectivity allow balanced networks to replicate the variability reported in both spontaneous and evoked conditions.

#### 2.2.1. Excitatory networks with clustered architectures

**Figure 2:**
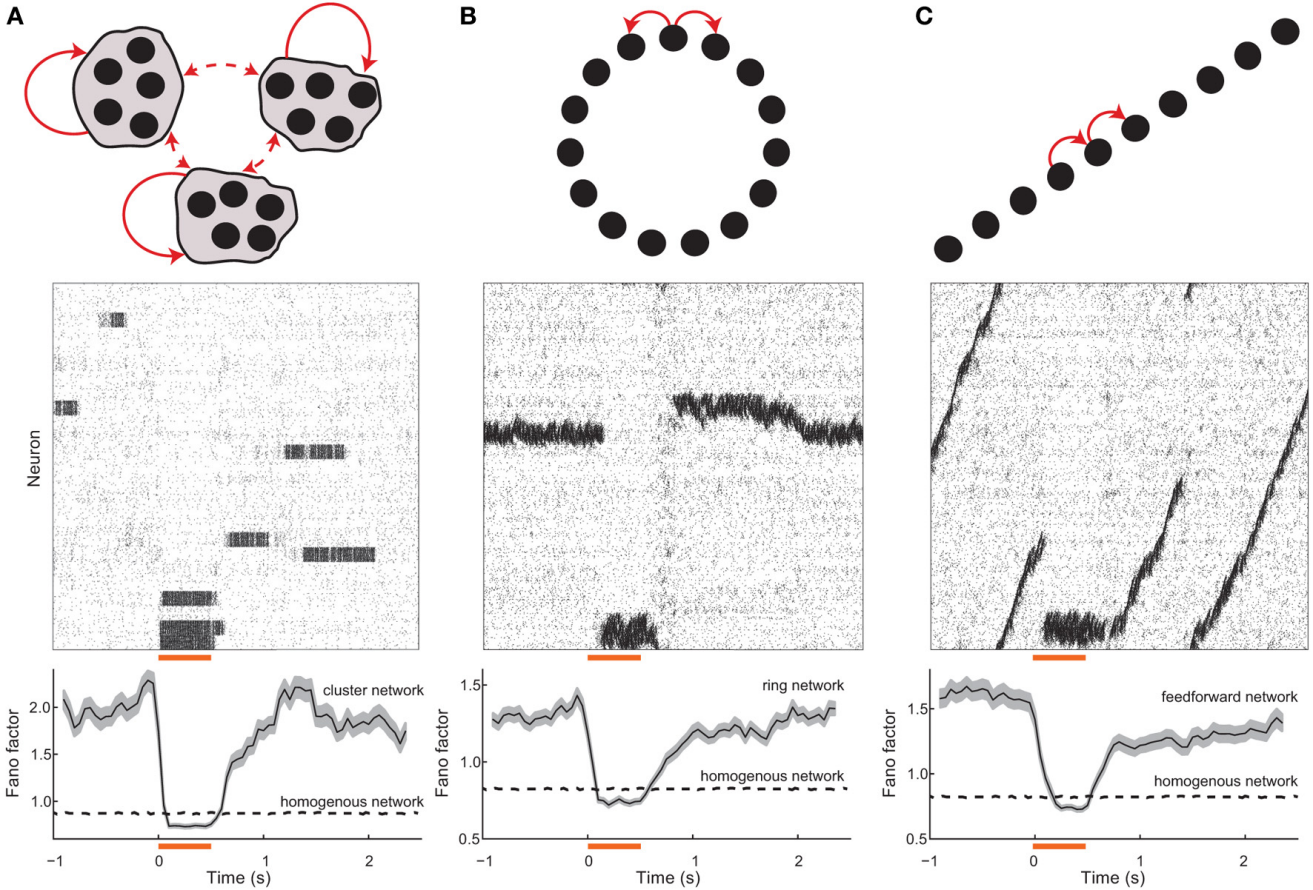
**Spontaneous and evoked variability in balanced networks with global architectures**. **(A)** (top) Schematic of a network with clustered excitatory connections. Solid (dashed) red arrows denote high (low) probability coupling. (middle) Spike train raster for the network; neurons are ordered with the cluster grouping. The orange bar denotes the stimulation period and the gray shaded region shows clusters that were stimulated. (bottom) Population averaged spike count Fano factor for the stimulated neurons; the shaded region denotes the standard error. **(B)** (top) Schematic of a network with ring topology. (middle) Spike train raster for the network; neurons are ordered with position on the ring. (bottom) Population averaged Fano factor for the stimulated neurons. **(C)** (top) Schematic of a network with dominant feedforward connections. (middle) Spike train raster for the network; neurons are ordered with position on the ring. (bottom) Population averaged Fano factor for the stimulated neurons. The Fano factor for a homogenous (i.e., unstructured) balanced network (see Figure 1) network is also shown (dashed lines). For the homogeneous networks a random subset of neurons were excited during the stimulation period. In all networks, the inhibitory connections were not selective for any excitatory topology, and rather connected to all targets with a uniform probability.

Over the past decade, several experimental groups have performed careful analysis of the microstructure in cortical networks. One clear finding is an architectural structure that is beyond that of a simple independent wiring model. In particular, small interconnected clusters of excitatory neurons are overrepresented in the cortex (Song et al., 2005; Perin et al., 2011). Cluster membership is often stimulus (Hofer et al., 2011; Ko et al., 2011), circuit input (Yoshimura and Callaway, 2005; Yoshimura et al., 2005), or activity (Yassin et al., 2010) dependent. Motivated by these anatomical findings, we subdivided excitatory cells into small clusters, and let the wiring probability be higher for two cells within the same cluster, compared to cells that are in distinct clusters (Figure 2A top, Materials and Methods). The chaotic dynamics of the network (Figure 2A, middle) is very distinct from that of an unclustered balanced network (Figure 1C). Specifically, cells within a cluster show periods of coordinated low and high firing activity, and the transitions between activity states occur over long timescales. These firing rate transitions are in addition to the spike time variability caused by the balanced state input fluctuations, so that spiking dynamics are “doubly stochastic.” Indeed, the spike count Fano factor over trials (normalized measure of variability, see Materials and Methods) is significantly above unity (Figure 2A, bottom), consistent with experimental measurements across cortex (Churchland et al., 2010).

When the neurons in a cluster are stimulated together they have a higher likelihood of being in a high firing rate state when compared to spontaneous conditions. Furthermore, recurrent inhibition permits only a fraction of the clusters to have high firing rates at any time, meaning that neurons in unstimulated clusters have a higher likelihood of being in a low firing rate state compared to spontaneous conditions. Thus, by stimulating a subset of the clusters, the network is biased to be in a specific firing rate configuration. This removes the trial-to-trial rate variability characteristic of spontaneous dynamics (Figure 2A, middle), leaving only the Poisson-like spike dynamics due to balanced excitation and inhibition. As a result the Fano factor is reduced to near one in evoked conditions (Figure 2A, bottom), again matching experiments (Churchland et al., 2010). In our analysis we have corrected for reductions in Fano factor that would be caused by an increase in firing rate. This was done through a mean-matching algorithm, originally presented in Churchland et al. (2010), that discards data in evoked and spontaneous conditions so that the firing rate distributions overlap (see Methods). Thus, the stimulus-induced reduction in Fano factor cannot be trivially due to an increase in the mean spike count that exceeds an increase in spike count variance, and is rather because of an active reduction in spike count variance.

The mean field analysis that was applied to unclustered balanced networks can be applied to clustered networks. Consider the mean synaptic input *M* given to a representative neuron. Split the recurrent excitation into the number of connections coming from within the neuron's cluster *K*_in_, and the remaining connections from outside the cluster *K*_out_ = *K* − *K*_in_. This gives the mean input as:

$$\begin{array}{l} M=\sqrt{K}\;\underset{O\left(1/\sqrt{K}\right)}{\underset{︸}{\left(j_{E}^{\text{out}}r_{E}^{\text{out}}-j_{I}r_{I}+i_{\text{ext}}\right)}}\\ \;+\underset{O(1)}{\underset{︸}{\left(K^{\text{in}}/\sqrt{K}\right)}}\underset{O(1)}{\underset{︸}{\left(j_{E}^{\text{in}}r_{E}^{\text{in}}-j_{E}^{\text{out}}r_{E}^{\text{out}}\right)}}\sim O(1). \end{array}$$

For excitatory cells that are within and outside the cluster we distinguish their connection strengths, *j*^in^_*E*_ and *j*^out^_*E*_, and firing rates, *r*^in^_*E*_ and *r*^out^_*E*_. The first term on the right hand side is *O*(1) when the large recurrent excitation from outside the cluster and external drive are balanced by large recurrent inhibition. This is identical to the balance condition of the unclustered network. The second term is a perturbation from the balanced state that is induced by the clustered architecture. For this term to neither vanish nor overwhelm the network we require that the number of inputs that drive a cell from within its cluster *K*^in^ scale as $\sqrt{K}$ (van Vreeswijk and Sompolinsky, 2005). For our network we set *K* ≈ 800 so that *K*^in^ ≈ 30, meaning that the excitation from within the cluster is a small fraction of the total recurrent excitation. Equivalently, for each neuron to be in a single cluster, we require the network to be divided into *Np*^in^_*EE*_/*K*^in^ ≈ 50 clusters. Overall, this means that the network will have a large number of stable states that provide a rich repertoire of dynamics in spontaneous conditions. The interested reader can see a full exposition of the trial-to-trial variability of balanced networks with clustered connections in Litwin-Kumar and Doiron (2012).

The above cluster-based mechanism for a stimulus-induced reduction in spiking variability suggests a general framework. Highly variable spontaneous dynamics should occur in balanced networks with a large number of symmetric stable firing rate configurations. When population driven fluctuations cause stochastic transitions between distinct rate configurations, individual neurons will inherit these transitions as a new source of long timescale firing variability. Stimuli can break the network symmetry and bias specific firing rate patterns, reducing trial-to-trial firing rate variability. As a proof of principle, we show stimulus-induced reductions in variability in balanced networks with two global architectures that are distinct from clustered networks, yet satisfy the core requirements of our framework.

#### 2.2.2. Excitatory networks with ring and feedforward architectures

Networks with local excitation and global (or lateral) inhibition arranged on a “ring” (Figure 2B, top) have been well studied as models of cortical orientation tuning (Ben-Yishai et al., 1995) as well as working memory (Compte et al., 2000). Ring networks support neutrally stable solutions where elevated rates are localized in space on the ring, often labeled a “bump” of activity (Ermentrout, 1998). In the absence of a stimulus, the network has a continuum of stable bump solutions, and population fluctuations allow a slow timescale wandering of the bump (Figure 2B, middle). Over realizations of the network, the random initial position and stochastic wandering of the bump drive slow firing rate variability so that single neuron Fano factors are large (Figure 2B, bottom). When a specific region on the ring is stimulated it becomes globally stable and other regions lose their stability, so that a bump of activity appears in that region and is pinned in space. Consequently, the firing rate variability is removed and single unit Fano factors drop to levels below spontaneous conditions (Figure 2B, bottom).

Another well studied architecture in theoretical neuroscience is networks with dominant feedforward coupling (Figure 2C top)^2^. Feedforward networks support the creation and propagation of synchronous waves of activity along the network (Kumar et al., 2010), as well as provide a mechanism for selective amplification of activity that produces long timescale dynamics (Goldman, 2009; Murphy and Miller, 2009). The symmetry in the network supports wave initiation at any position in the chain, so that population fluctuations spuriously cause and destroy wave dynamics over time (Figure 2C, middle). Over trials of the network, these wave events drive single unit firing rate variability so that Fano factors are elevated (Figure 2C, bottom). Stimuli at a fixed point along the chain break the translational symmetry along the network and effectively pin activity in that region since global network inhibition prevents wave propagation. Thus, over repeated trials the wave induced dynamics are absent and the evoked Fano factor are below what is observed in spontaneous conditions (Figure 2C, bottom).

In total, networks with balanced excitation and inhibition with ring or feedforward architectures also capture high variability in spontaneous conditions and the reduction of variability in evoked states.

### 2.3. Coherence between stimulus drive and recurrent architectures

**Figure 3:**
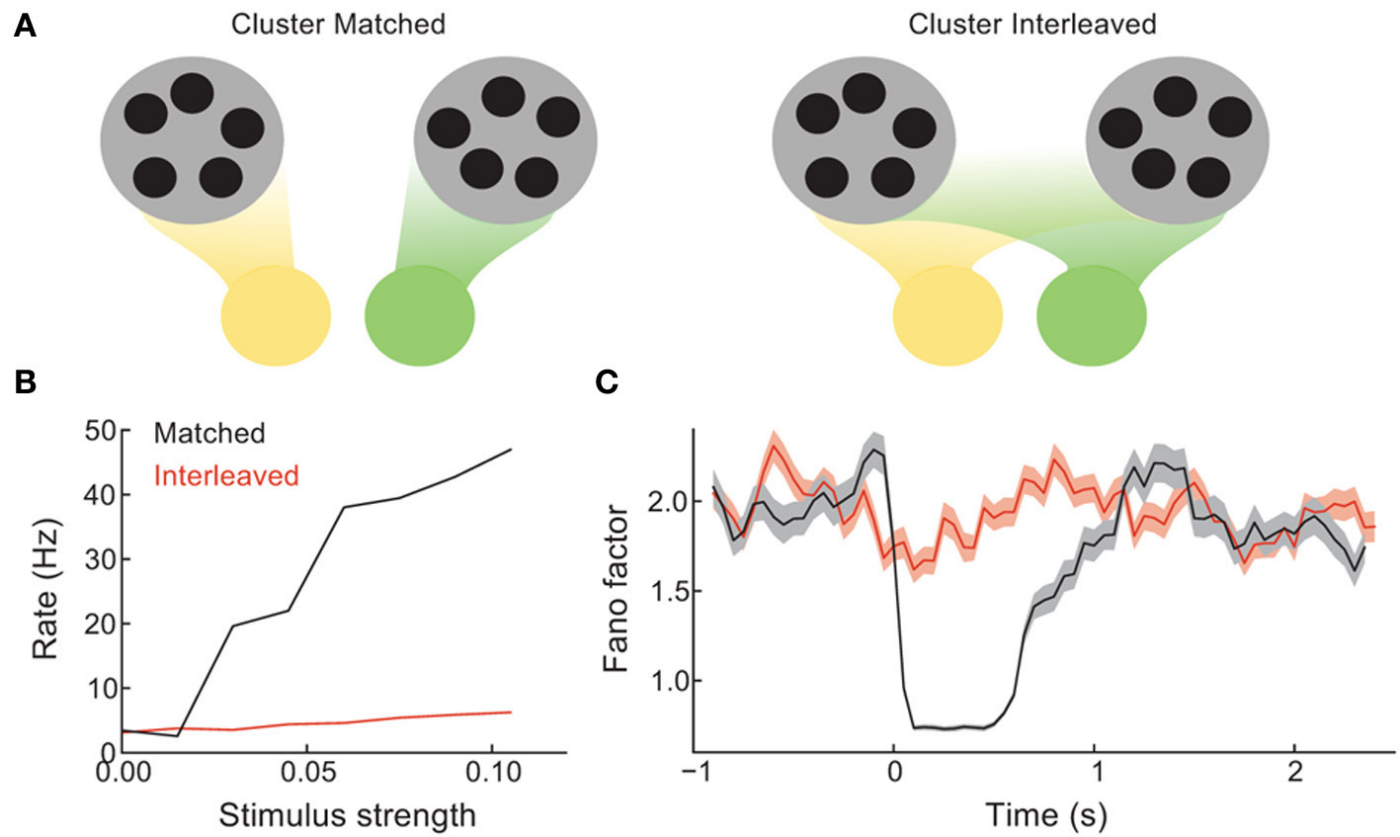
**Coherence between the spread of stimulation over the network and the recurrent architecture controls the difference between spontaneous and evoked activity**. **(A)** Schematic of the case when distinct stimuli (green vs. yellow) drive either distinct clusters (left) or are distributed evenly over all clusters (right). **(B)** The mean trial-averaged firing rate of a stimulated neuron as a function of stimulus amplitude. **(C)** The spike count Fano factor during the time course of spontaneous and evoked states.

In the balanced network with clustered excitation, we chose feedforward stimulus drive to be aligned with cluster membership (Figure 2). In other words, neuron pairs that belonged to the same cluster received coordinated stimulation, while neuron pairs in different clusters did not. In this section we compare the recruitment of clustered network activity when stimulation respects cluster membership (cluster matched, Figure 3A, left) to when a random assortment of neurons are stimulated, with no relation to the underlying cluster membership (cluster interleaved, Figure 3A, right).

The stimulus-response gain of the trial-averaged single neuron firing rate is higher for the cluster matched than for the cluster interleaved protocol (Figure 3B). Furthermore, the cluster interleaved stimulation did not cause a reduction in the spike count Fano factor compared to the spontaneous state, in contrast to the cluster matched case (Figure 3C). Thus, for the stimulus intensities studied, only cluster matched stimulation cause an evoked response that differs from the spiking statistics of the spontaneous state. This result is expected, as we describe below.

By design, the cluster of neurons driven by cluster matched inputs share more recurrence with one another than the neurons driven by cluster interleaved inputs. Because of this the positive feedback recruited by the stimulus is larger for the cluster matched protocol than for the interleaved protocol. Positive feedback increases response gain in spiking networks (Sutherland et al., 2009), allowing the response to weak inputs to be amplified. Furthermore, when the stimulation is interleaved, each cluster receives inputs to only a fraction of its neurons, distributed roughly evenly over the clusters. Thus, stimulus interleaved stimulation does not bias any one firing rate configuration more than any other, and the mechanism for firing rate variability is left unaffected. Hence so that the Fano factor remains high throughout stimulation. In total, these results show that a coherence between how the stimulus is distributed over the network and the global recurrent architecture is needed for evoked spiking variability to differ from those in the spontaneous state.

### 2.4. Relating spiking correlations in the spontaneous and evoked states

**Figure 4:**
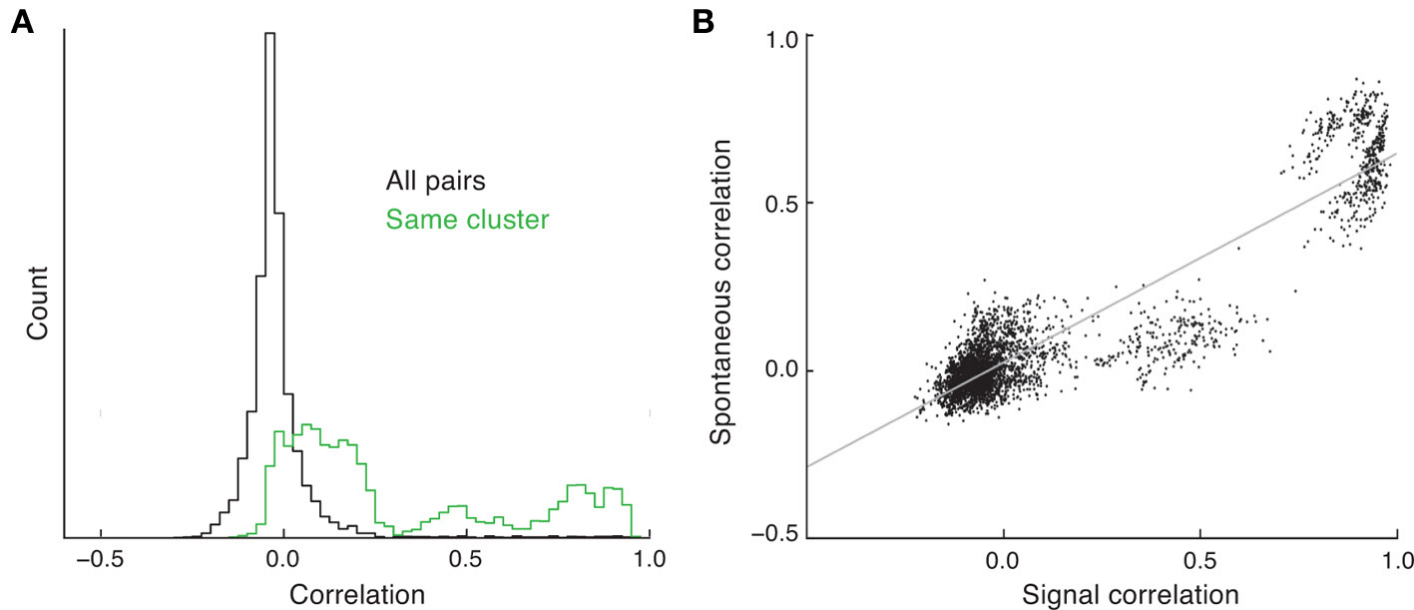
**Spike count correlations in clustered networks**. **(A)** Estimated density of spike count correlation coefficients for all pairs (black) and pairs that are in the same cluster (green). **(B)** The relation between signal and spontaneous correlations in the clustered network.

Balanced networks with unstructured connectivity have a stable asynchronous state (Renart et al., 2010). As such, in these networks the spike count correlation coefficient from pairs of neurons is on average close to zero, and for large networks (*N* > 1000) the likelihood of correlation coefficients above 0.2 is negligible. The clustered network is also balanced and, similar to the unclustered network, has a density of pairwise correlation that is approximately Gaussian with a small spread about a near zero mean (Figure 4A, black curve). While asynchronous dynamics are consistent with some cortical recordings (Ecker et al., 2010; Renart et al., 2010), there are many studies that show an average positive spike count correlation, with some pairs having significant (>0.2) correlations (Cohen and Kohn, 2011). However, in these experimental studies the correlation is measured from evoked, and not spontaneous, states.

The trial-to-trial co-variability of pairwise response in evoked states is termed “noise” correlations (Averbeck et al., 2006), to be distinguished from correlations in the trial-averaged activity due to the signal preferences of the neuron pair, i.e., “signal” correlations. In the clustered network we have assumed that stimulus preference and cluster membership are related (Figure 3). When pairwise correlation is conditioned on each neuron of the pair being a member of the same cluster, the mean correlation is far from zero, and the density has a heavy tail at positive correlations (Figure 4A, green curve). This is due to neurons that are members of the same cluster being subject to the same firing rate fluctuations in spontaneous conditions, manifesting as a positive correlation coefficient. Thus, clustered architectures in balanced networks provide a mechanism for positive spike count correlations between select pairs. Further, because of the large number of clusters in the network, the number of pairs that are in the same cluster is far less than the number of total pairs in the network. This fact allows the clustered architecture to support a rough asynchronous state across the entire network, a requirement of balanced solutions (Renart et al., 2010).

The combination of these results suggests that a pair of neurons in the same cluster will have both a net positive correlation and an assumed common feedforward stimulus drive. This produces a clear and testable prediction: the signal correlation between pairs of neurons should be positively related to their correlation in the spontaneous state. This is certainly the case in our network simulations (Figure 4B); however, this has yet to be tested from *in vivo* data (to our knowledge).

### 2.5. Spontaneous activity “samples” the range of evoked states

Our analysis of spontaneous and evoked network activity has focused on its reflection in single neuron (Figures 2, 3) or pairwise (Figure 4) spike count statistics. However, the high correlation for pairs of neurons within the same cluster suggests that an analysis of the population dynamics of pairs of clusters will provide a network perspective on the dynamics of spontaneous and evoked activity.

**Figure 5:**
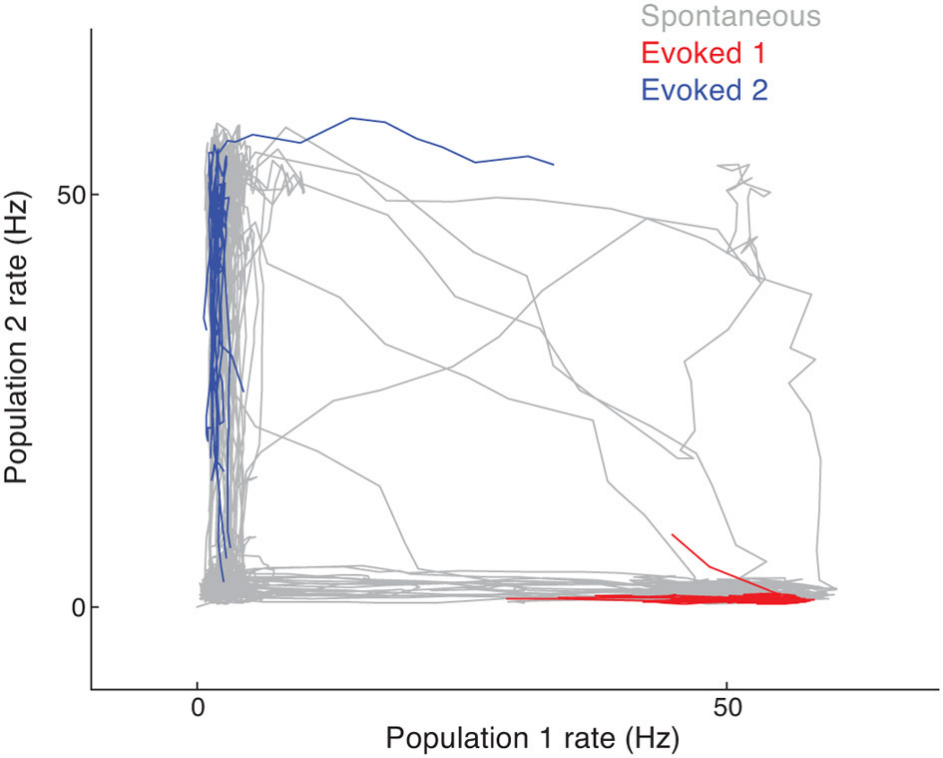
**Joint firing rate trajectory in spontaneous and evoked conditions for the clustered network**. The gray curve is the trajectory during spontaneous activity, while the red and blue curves are for stimulation of the first and second cluster, respectively.

In the spontaneous state, cluster activity randomly transitions between low and high firing rate regimes. The recurrent inhibition regulates the overall network activity such that if neurons in one cluster are firing at high rates then neurons in another cluster are likely firing at low rates. This is seen in the joint activity of a pair of clusters in the network, since their joint firing rate trajectory shows an anti-correlation (Figure 5, gray curve). However, when a stimulus is given that drives either one of the clusters, the joint firing rate trajectory is effectively clamped so that the driven cluster's firing rate is high and the other cluster's is low. In the space of joint firing rates this collapse of network dynamics is such that the evoked state is a subset of the spontaneous state (Figure 5, red and blue curves compared to the gray curves).

### 2.6. Stimulus quenched variability in chaotic firing rate networks

We have presented a model for spontaneous cortical dynamics with high variability that is based on either a discrete (Figure 2A) or continuous (Figures 2B,C) attractor structure. Attractor dynamics require that recurrent cortical architecture provides a large set (or continuum) of stable network states. Such a constraint confers a fragility to the model dynamics, with unstructured heterogeneity in network architecture causing a collapse of network dynamics to only a few stable states. This criticism of attractor dynamics is often countered by assuming appropriate homeostatic mechanisms which provide a robustness to the attractor structure (Renart et al., 2003; Vogels et al., 2011). However, more research is needed to properly understand how attractor dynamics depends on the combination of homeostasis and rich spontaneous dynamics.

An alternative model of cortex is a recurrent excitatory–inhibitory network built from phenomenological firing rate models (Ermentrout and Terman, 2010). When recurrent coupling is strong, these models show chaotic solutions (Sompolinsky et al., 1988; Sussillo and Abbott, 2009; Rajan et al., 2010), where firing dynamics are trial-to-trial variable due to the extreme sensitivity of the state of the network at the time of stimulation. In contrast to attractor networks, these networks are not dependent on any fine structured architecture, so that the chaotic solutions are robust to parameter variations. However, by design these models consider only output firing rates, and we must assume a suitable mechanism for how spike trains are generated from a rate dynamic. It is natural to consider the rate solutions as generating an inhomogeneous Poisson process. In this case, the trial variable chaotic dynamics of the rate model would produce spike trains that are “doubly stochastic” point processes, with an expected Fano factor larger than unity (Churchland and Abbott, 2012).

**Figure 6:**
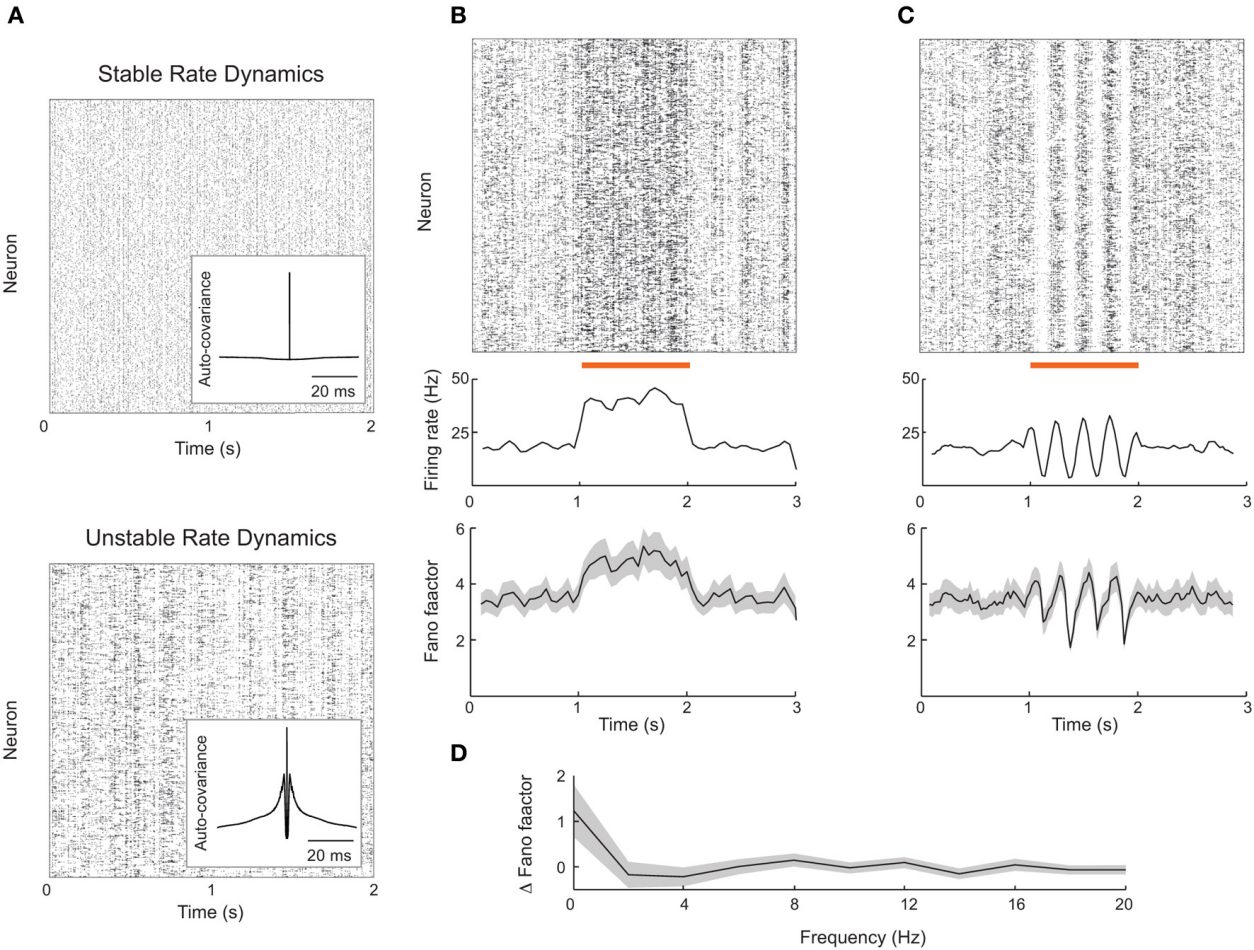
**Firing rate instabilities in spiking networks do not show stimulus-induced quenching of trial-to-trial variability**. **(A)** Spike train rasters from a recurrent excitatory–inhibitory network in stable firing rate (top) and unstable firing rate (bottom) regimes. **(B)** Spike count mean and Fano factor during spontaneous and evoked (zero-frequency stimulus) conditions. **(C)** Same as **(B)**, but with periodic stimulation. **(D)** The change in Fano factor Δ *F* = *F*_*spont*_ − *F*_*evoked*_ as a function of stimulation frequency. Here Fano factors are time averaged during stimulation. All Fano factors were computed with mean matched data. Network parameters are identical to those of Ostojic (2014) with coupling strength *J* = 0.2 (stable network) or *J* = 0.6 (unstable network). Stimuli consisted of a modulation of the external bias to half of the 8000 excitatory neurons by an amount given by 0.25 cos(2π*ft*). All Fano factors were computed using a mean-matched analysis.

Rajan et al. (2010) observed that global, dynamic stimulation quenched internally generated chaotic dynamics in firing rate models. Thus, network firing rates in stimulated conditions lacked the dynamic variability characteristic of the spontaneous state. If a network produced spikes according to an inhomogenous Poisson process with firing rates consistent with this model, then we expect a stimulus-induced reduction in the Fano factor, similar to that reported in our attractor model (Figure 2). However, it is still unclear if a network composed of actual spiking units, rather than interacting firing rates, is capable of reproducing the results of Rajan et al. (2010). This reproduction is not trivial because in most recurrent networks firing rates are stable (Figure 6A, top) (van Vreeswijk and Sompolinsky, 1996, 1998; Brunel, 2000; Renart et al., 2010), as opposed to chaotic.

Fortunately, Ostojic (2014) has recently shown how strong recurrent coupling in excitatory–inhibitory networks of spiking neuron models can destabilize firing rates, causing chaotic dynamics reminiscent of those predicted in firing rate networks (Figure 6A, bottom). This destabilization produces longer timescale spike train fluctuations reminiscent of a burst-like discharge that is absent in standard balanced networks (compare inset auto-covariance functions in Figure 6A). The network nevertheless shows roughly asynchronous spiking behavior and is a reasonable candidate model for rich spontaneous cortical dynamics. We implemented this spiking network in an effort to test the alternative theory for stimulus induced reduction in spike count variability proposed by Rajan et al. (2010).

The rate variability in the strongly coupled network produces high trial-to-trial variability in spiking dynamics in the spontaneous state, as characterized by a sizable Fano factor (~4; Figure 6B, bottom panel before stimulation). When a static depolarizing input stimulus was applied to a half of the excitatory neurons, the firing rates of the stimulated neurons naturally increased (Figure 6B, top and middle panels). However, the mean matched Fano factor also increased, because the elevated firing rates recruited more burst behavior (Figure 6B, bottom panels). This is in contrast to the drop in variability seen in the attractor networks presented earlier (Figure 2), as well recorded spike trains from sensory and motor cortex (Churchland et al., 2010).

We also tested if periodic stimulation could quench the spiking variability in strongly coupled networks, as suggested by chaotic rate networks (Rajan et al., 2010) as well as data from rodent visual cortex (White et al., 2012). Periodic input was reflected in a periodic modulation of both firing rate and spike count Fano factor (Figure 6C); however, the average Fano factor was not reduced by stimulation. This weak modulation of Fano factor, despite large changes in evoked rates, occurred over a range of stimulation frequencies (Figure 6D). Thus, further work is needed to determine whether certain stimulus or network structures can reproduce the quenching of chaos seen in continuous firing rate networks.

## 3. Discussion

The relation between spontaneous and evoked cortical dynamics is a popular topic of study (Ringach, 2009). Voltage sensitive dye imaging of visual cortex in cats has shown the spontaneous dynamics of large regions of cortex can mirrors evoked dynamics (Arieli et al., 1996; Tsodyks et al., 1999; Luczak and MacLean, 2012). Population recordings in the auditory system of rodents (Luczak et al., 2009) and primates (Fukushima et al., 2012) show that spontaneous dynamics contain a large repertoire of activity, with an evoked state being merely a subset of that repertoire. Our study contributes to this literature by identifying cortical network architecture which supports rich spontaneous dynamics, that nevertheless has a clear relation to evoked dynamics. We expand on our previous study (Litwin-Kumar and Doiron, 2012) and argue that the excess variability in spontaneous conditions (Churchland et al., 2010) is a characteristic of balanced networks with a large number of stable firing rate states.

### 3.1. Balanced attractor networks and scaling

The networks discussed in this paper are a merging of ideas from the balanced network literature (van Vreeswijk and Sompolinsky, 1996, 1998; Renart et al., 2010), which proposes models of trial-to-trial variability, and the Hopfield network literature (Hopfield, 1982), which proposes models of neuronal assemblies. This connection has been made in several past studies (Amit and Brunel, 1997; van Vreeswijk and Sompolinsky, 2005; Renart et al., 2007; Roudi and Latham, 2007). Our contribution is specifically to study the implications of such attractor structures for trial-to-trial variability. Below, we discuss more thoroughly the plausibility of merging these ideas in the context of scaling arguments (see Renart et al., 2007 for an additional discussion of balance and scaling).

One requirement needed to merge these two lines of research was a specific scaling of the size of the neuronal assemblies. In balanced networks, if neurons receive *K* connections, then the connection strength *J* must be proportional to 1/$\sqrt{K}$. We considered the case of *dense connectivity*, so that *K* ∝ *N*. Further, the perturbation away from a homogeneous balanced network due to the attractor structure was set to be *O*(1), to prevent large attractors from dominating the network activity. Let the size of a neuronal assembly be *K*^in^ neurons and define the *coding fraction* as *K*^in^/*N*. Then we must have:

$$K^{\text{in}}(J_{E}^{\text{in}}p^{\text{in}}-J_{E}^{\text{out}}p^{\text{out}})\sim O(1),$$

where *J*^in^_*E*_ − *J*^out^_*E*_ is how much stronger connections within the assembly are and *p*^in^ − *p*^out^ is how much more probable they are. There are three scenarios:

1. *K*^in^ ∝ *K*, that is, each assembly is composed of a macroscopic number of neurons and the coding fraction is constant. This is the case of a classic Hopfield network (Hopfield, 1982). However, to satisfy Equation (1), we must have *J*^in^_*E*_ *p*^in^ − *J*^out^_*E*_ *p*^out^ ∝ 1/*K*. This presents a fine-tuning problem, as the *J*'s themselves are proportional to 1/$\sqrt{K}$, much larger than the desired difference between *J*^in^_*E*_ and *J*^out^_*E*_. Classic Hopfield networks avoided this problem as *J* was proportional to 1/*K*.
2. *K*^in^ ∝ $\sqrt{K}$, so the coding fraction is small. This was the case considered in our study and does not require fine-tuning as setting *J*^in^_*E*_ = *aJ*^out^_*E*_ for some constant *a* ensures Equation (1) is satisfied. In such networks, the number of neurons in a local assembly is smaller by a factor of $\sqrt{K}$ than the total number of inputs received. If stimuli are assumed to excite a few assemblies at once, this corresponds to a network exhibiting *sparse coding*, which is frequently encountered in cortex (Hromádka et al., 2008).
3. *K*^in^ ∝ 1. This corresponds to a case of extremely sparse coding, as assembly size does not grow at all with *K*. Further, in this case we must have *J*^in^_*E*_ = *O*(1) while *J*^out^_*E*_ = *O*(1/$\sqrt{K}$), corresponding to a few extremely strong local connections. For these reasons, we do not consider this case.

Typically, spiking implementations of attractor networks fall into the second category (Amit and Brunel, 1997; van Vreeswijk and Sompolinsky, 2005; Renart et al., 2007), as was true for our study. Roudi and Latham (2007) took an alternative approach and studied the first category, differentiating between “background weights” that were *O*($\sqrt{K}$) and “foreground weights” (connections between neurons in the same assembly) that were *O*(1/*K*). In order to obtain stable assembly activation, these foreground weights needed to be tuned to within 6% in a network of 10,000 neurons, and it is unclear that such fine-tuning is plausible.

It is therefore an open question how to robustly merge balanced and attractor networks with a non-vanishing coding fraction. Unlike primary sensory regions, higher-order associative regions such as prefrontal cortex have a large degree of “mixed selectivity,” with high coding fractions and assembly overlap (Rigotti et al., 2013). The fine-scale architecture of such regions has yet to be determined.

### 3.2. Symmetric and finite size clusters

Despite their name, balanced networks are *robust* to changes in recurrent synaptic strengths *J* and admit a stable asynchronous solution over a wide range of network firing rates (van Vreeswijk and Sompolinsky, 1996, 1998; Renart et al., 2010). However, the mechanism for spontaneous dynamics presented in this study requires a strong assumption of symmetry in the network architecture. More to the point, rich spontaneous dynamics requires that each firing rate configuration be roughly equally stable. If this is not the case then some stable configurations will be over-represented in the spontaneous state, and the population firing rates will tend to reside in those states. In the limit where one specific state is very stable, then the spiking variability in spontaneous and evoked dynamics would be similar. In our model, symmetric metastability is achieved by assuming that all clusters are of the same size, introducing a certain fragility into the framework. This is not an exact symmetry since quenched connection variability is present in any realization of the network. Nevertheless, our framework cannot support clusters of widely different sizes, as might be expected in real cortical networks. Future work should leverage powerful homeostatic mechanisms (Turrigiano and Nelson, 2000; Vogels et al., 2011) to prevent “winner-take-all” dynamics when the size and recurrence are heterogeneous across attractors.

The fluctuations in our model are an emergent feature of recurrent wiring, as opposed to being imposed by an external source. Balanced networks have a well defined solution as the number of inputs *K* → ∞ (van Vreeswijk and Sompolinsky, 1996, 1998; Renart et al., 2010). However, in the clustered network the internal variability that promotes stochastic transitions between stable firing rate states requires *K* < ∞. Otherwise the time between firing rate transitions diverges, and the spontaneous state is no longer dynamically rich. Estimates for the number of neurons that are afferent to a cortical cell are imperfect, since anatomically and functionally defined architectures are often distinct, making a concrete value for *K* is difficult to obtain. Further, estimates for the size of a cluster are also difficult; however, (Perin et al., 2011) give an indirect number of ~100 neurons per cluster, consistent with our network model. For models of cortical networks to improve, techniques for functional microcircuit measurements of large numbers of cells must be developed.

### 3.3. Cortical architecture determines variability in spontaneous and evoked states

In our model, feedforward afferent projections are assumed to be coherent with recurrent architecture (Figure 3A). Without this feature stimulus drive does not quench trial-to-trial variability (Figure 3C), and our model would then be at odds with experimental data across sensory and motor cortex (Churchland et al., 2010). Also, if stimulus input recruited distinct populations than those recruited during spontaneous activity, then the patterning of spontaneous activity would not resemble that in evoked states, again in disagreement with cortical data (Tsodyks et al., 1999; Luczak et al., 2009). This circuit assumption is well justified from recent circuit recordings in mouse visual cortex (Hofer et al., 2011; Ko et al., 2011), where the stimulus correlation and connection probability of pairs of neurons are positively correlated with one another. Thus, a specific stimulus should drive neurons that are members of a specific cluster to a larger degree than other neurons in the network. Finally, such a wiring rule is consistent with standard Hebbian plasticity rules, where repeated co-activation of neuron pairs would engage plasticity rules that strengthen recurrent coupling between them.

Understanding the trial-to-trial variability in evoked conditions is important for a complete description of a population code (Dayan and Abbott, 2001; Averbeck et al., 2006; Josić et al., 2009; Ponce-Alvarez et al., 2013). For this reason, a large number of studies focus on the trial-to-trial “noise” correlations of neuron pairs in evoked states (Cohen and Kohn, 2011). In both visual (Smith and Kohn, 2008; Ponce-Alvarez et al., 2013) and auditory (Rothschild et al., 2010) cortex, signal and noise correlations are themselves correlated, so that neurons that have similar stimulus preference, i.e., positive signal correlation, also have positive noise correlation. This suggests that the underlying circuits that establish stimulus preference also contribute to common fluctuations. Our model extends this idea to the spontaneous state, and predicts a positive relation between signal correlations and the correlations measured during spontaneous conditions. However, balanced networks (including the ones used in this study) fail to capture the positive average pairwise correlation that is consistent with some (Cohen and Kohn, 2011), yet not all (Ecker et al., 2010), data sets (Figure 4A, black curve). A spiking network framework that internally generates non-zero mean correlation consistent with data is, to our knowledge, an open problem.

In sum, our model shows that a coherence between feedforward and recurrent architectures produces cortical dynamics that is consistent with a wide array of spontaneous and evoked data, and makes a clear and testable prediction for pairwise correlations in the spontaneous state.

## 4. Materials and methods

### 4.1. Spiking network simulations

Neurons were modeled as leaky integrate-and-fire units whose voltages obeyed:

$$\dot{V}=\frac{1}{\tau }\left(\mu -V\right)+I_{syn}(t)$$

When neurons reached a threshold *V*_*th*_ = 1, a spike was emitted and they were reset to *V*_*re*_ = 0 for an absolute refractory period of 5 ms. The membrane time constant τ was 15 and 10 ms for excitatory and inhibitory neurons, respectively. The bias μ was chosen according to a uniform random distribution between 1.1 and 1.2 for excitatory neurons and between 1 and 1.05 for inhibitory neurons. While these values are superthreshold, balanced dynamics ensured that the mean membrane potentials were subthreshold (van Vreeswijk and Sompolinsky, 1998). In Figure 1, the non-dimensionalized voltages were transformed so that *V*_*th*_ = −50 mV and *V*_*re*_ = −65 mV.

Synapses between neurons were modeled as differences of exponentials, and the total synaptic input to neuron *i* in population *x* was:

$$I_{i,syn}^{x}(t)=\sum_{jy}J_{ij}^{xy}F^{y}*s_{j}^{y}(t)$$

where * denotes convolution. Here *x*, *y* ∈ {*E*, *I*} denote excitatory or inhibitory populations of *N*^*E*^ = 4000 and *N*^*I*^ = 1000 neurons each, *J*^*xy*^_*ij*_ is the strength of synaptic connections from neuron *j* in population *y* to neuron *i* in population *x*, *F*^*y*^ (*t*) is the synaptic filter for projections from neurons in population *y*, and *s*^*y*^_*j*_ (*t*) is the spike train of neuron *j* in population *y*, a series of delta-functions at the time points where the neuron emitted a spike. *F*^*y*^ (*t*) = $\frac{1}{\tau_{2}-\tau_{1}}$ (*e*^−*t*/τ_1_^ − *e*^−*t*/τ_2_^), with τ_2_ = 3 ms for excitatory synapses and 2 ms for inhibitory synapses while τ_1_ = 1 ms.

Connection probabilities *p*^*xy*^ from neurons in population *y* to *x* were *p*^*EI*^ = *p*^*IE*^ = *p*^*II*^ = 0.5, while *p*^*EE*^ was on average 0.2. In clustered networks, excitatory neurons were partitioned into 50 assemblies of 80 neurons each and the connection probability was set to *p*^*EE*^_in_ for neurons in the same assembly and *p*^*EE*^_out_ for neurons in different assemblies. These were chosen so that *p*^*EE*^ remained on average 0.2, but *p*^*EE*^_in_/p^*EE*^_out_ = *R*^*EE*^ = 2.5. For ring and feedforward networks, periodic boundary conditions were applied by identifying *i* = 4000 with *i* = 0. For ring networks, neurons *i* and *j* were said to belong to the same assembly if |*i* − *j*| < 40. For feedforward networks, connections from neuron *i* to neuron *j* were drawn with probability *p*^*EE*^_in_ if *i* − *j* ∈ [−35, 45] and *p*^*EE*^_out_ otherwise, thus biasing connections in one direction along the ring. If a connection from neuron *j* in population *y* to neuron *i* in population *x* existed, *J*^*xy*^_*ij*_ = *J*^*xy*^ (unless *x*, *y* = *E* and neurons were in the same assembly, then connection strength was multiplied by 1.9), otherwise *J*^*xy*^_*ij*_ = 0. Synaptic strengths were *J*^*EE*^ = 0.024, *J*^*EI*^ = −0.045, *J*^*IE*^ = 0.014, and *J*^*II*^ = −0.057. These parameters, multiplied by 15 mV, would give the deflection of the membrane potential of the post-synaptic target, neglecting leak, in our dimensionalized units. When clusters of excitatory neurons were stimulated, stimulation was accomplished by increasing μ for neurons in those clusters by 0.07. Simulations were performed using Euler integration with a timestep of 0.1 ms.

### 4.2. Spike train statistics

Spike train statistics were computed for excitatory neurons. We denote the spike times of neuron *i* as {*t*_*i*1_, *t*_*i*2_, *t*_*i*3_, …}. We can then define neuron *i*'s spike train: *y*_*i*_ (*t*) = ∑_*k*_ δ (*t*_*ik*_). The number of spikes emitted by the neuron between times *t* and *t* + Δ *t* is

$$N_{i}(t,t+\Delta t)=\int_{t}^{t\;+\;\Delta t}y_{i}(t^{\prime })dt^{\prime }$$

The firing rate of a neuron over an interval (*t*, *t* + Δ *t*) was defined as

$$r_{i}(t,t+\Delta t)=\frac{1}{\Delta t}N_{i}(t,t+\Delta t)$$

For the networks studied, firing rates and other statistics for the spontaneous state were calculated with *t* = 1.5 s to prevent effects due to initial conditions and Δ *t* = 1.5 s.

We also computed the Fano factor *F*_*i*_ (*t*, *t* + Δ *t*) for neuron *i* by evaluating

$$F_{i}(t,t+\Delta t)=\frac{\text{Var}\left(N_{i}(t,t+\Delta t)\right)}{\langle N_{i}(t,t+\Delta t)\rangle }$$

where the expectations are over repeated trials of the same network with random initial conditions. When computing the Fano factor as a function of time relative to stimulus onset, we computed the mean-matched Fano factor described in Churchland et al. (2010) to control for changes in firing rate. Fano factors were computed over 100 ms windows.

We computed correlation coefficients for the spike counts of neuron pairs. The correlation between neurons *i* and *j* was given by

$$\rho_{ij}=\frac{\text{Cov}\left(N_{i}(t,t+\Delta t),N_{j}(t,t+\Delta t)\right)}{\sqrt{\text{Var}\left(N_{i}(t,t+\Delta t)\right)\text{Var}\left(N_{j}(t,t+\Delta t)\right)}}$$

where the covariances and variances were computed over overlapping windows within each trial and then averaged across trials. For spontaneous activity trial averaging was replaced with temporal averaging.

## Funding

NSF-DMS1313225 and NSF-DMS1121784.

### Conflict of interest statement

The authors declare that the research was conducted in the absence of any commercial or financial relationships that could be construed as a potential conflict of interest.

## Footnotes

^1^However, in these studies a fine balance between excitation and inhibition is required, necessitating some fine tuning of network parameters.

^2^In our networks we did not force a strict feedforward structure, instead we introduced an anisotropy along the chain so that backward connections were less likely than forward connections.
